# Incidence of cancer in chronic inflammatory demyelinating polyneuropathy: a nationwide cohort study in South Korea

**DOI:** 10.3389/fneur.2024.1456835

**Published:** 2024-08-29

**Authors:** Kyomin Choi, Sohee Jung, Gucheol Jung, Dayoung Kim, Jeeyoung Oh

**Affiliations:** ^1^Department of Neurology, College of Medicine, Soonchunhyang University Cheonan Hospital, Cheonan-si, Republic of Korea; ^2^Department of Medical Artificial Intelligence, Deepnoid Inc., Seoul, Republic of Korea; ^3^Department of Neurology, College of Medicine, Konkuk University Medical Center, Seoul, Republic of Korea

**Keywords:** chronic inflammatory demyelinating polyneuropathy, incidence, neoplasms, cancer, health insurance

## Abstract

**Background:**

Chronic inflammatory demyelinating polyneuropathy (CIDP) is a rare disease, and the potential risk of cancer in patients with CIDP remains an important concern during treatment. However, a comprehensive epidemiological study examining this association is yet to be conducted. This study aimed to investigate the incidence of cancer in patients with CIDP in South Korea using data from the Korean Health Insurance Review and Assessment Service (HIRA) database.

**Methods:**

Data from the HIRA database between January 2016 and June 2021 were analyzed. The actual incidence of cancer in patients with CIDP was compared with the expected incidence based on the general population statistics in South Korea, with adjustments for age.

**Results:**

In total, 888 patients with CIDP were included in the analysis, of whom 50 (5.63% of malignancy incidence) were newly diagnosed with cancer during the study period. Among the patients with CIDP diagnosed with cancer, 32 (64.00%) were aged 60 years or older, and 36 (72.00%) were male. The observed number of cancer diagnoses corresponded to an incidence rate of 5.63%, with a standardized incidence ratio (SIR) of 2.83 (95% confidence interval [CI]: 1.89–4.39) compared to the expected cancer incidence rate of 2.00%. Notably, the SIR for malignancies of lymphoid, hematopoietic, and related tissues, excluding malignant immunoproliferative diseases, multiple myeloma, and plasma cell neoplasms (C81-96, except C88 and C90), was the highest at 8.51 (95% CI: 4.18–19.83).

**Conclusion:**

Our study shows a potential association between CIDP and an increased risk of hematological malignancies, which is consistent with previous investigations. Further studies are required to better understand the relationship between CIDP and cancer.

## 1 Introduction

Chronic inflammatory demyelinating polyneuropathy (CIDP) is a rare immune-mediated disorder that affects the peripheral nerves and nerve roots (1). The disease has a variable course and requires long-term treatment, often involving the adjustment of immunosuppressive medications (2). Cancer risk can be a concern in CIDP treatment because patients with CIDP are exposed to several risk factors for malignancy such as physical inactivity, malnutrition, and infection (3, 4). Moreover, lifelong immunosuppressive treatment potentially increases cancer risk (5). Several factors can alter the risk of cancer, and the relationship between CIDP and cancer is complex. A systematic review conducted in 2018 reported that hematological disorders and melanoma are common malignancies in patients with CIDP (6). However, the enrolled studies were small case series and isolated case reports, as there is insufficient epidemiological information on CIDP (6).

In South Korea, a public medical insurance system known as the National Health Insurance (NHI) covers nearly the entire population (7, 8). Under the NHI, the Health Insurance Review and Assessment Service (HIRA) collects data on patient diagnoses, treatments, procedures, and prescription drugs to evaluate medical fees, quality of care, and adequacy of medical services. The HIRA database has been used in several epidemiological studies to evaluate the cancer risk in specific medical conditions (9–11).

This study aimed to estimate the cancer incidence rates in patients with CIDP and compare them with those of the general population, as measured by Statistics Korea, a government organization. Based on a previous systematic review (6), we hypothesized that the incidence of hematological and dermal malignancies would be elevated in patients with CIDP, whereas the incidence of other cancers would not differ from that of the general population.

## 2 Methods

### 2.1 Data source

The public medical insurance system in South Korea, known as the NHI Service, covers approximately 98% of the South Korean population, as South Korean law requires every resident to be covered under this scheme (12). All medical processes are conducted under patients’ diagnostic codes of the Korean Standard Classification of Disease (KCD) and the Korean version of the International Classification of Diseases. The HIRA claims data include patient age, sex, diagnoses, medical costs, procedures, prescribed drugs, and unique anonymous numbers for each patient (7, 12).

### 2.2 Study design and population

This retrospective population-based cohort study utilized the HIRA data to identify patients with CIDP. Both diagnostic codes and medication histories were used to identify patients. First, patients with CIDP were identified using a primary diagnostic code of G61.8 according to the 7th and 8th revisions of the KCD (KCD-7, KCD-8), from 1st January, 2016 to 30th June, 2021. The G61.8 code refers to “other inflammatory neuropathies,” includes both CIDP and multifocal motor neuropathy (MMN). To qualify for reimbursement from the NHI Service, South Korean medical institutions should diagnose CIDP according to the criteria of the European Federation of Neurological Societies/Peripheral Nerve Society from 2010 (13). Second, a history of immunosuppressive treatment or steroid for at least 3 months between 1st January, 2016 to 30th June, 2021 was required to exclude MMN. Regular intravenous immunoglobulin therapy is recommended as initial and maintenance treatment for MMN (2). This immunosuppressive maintenance treatment should be adjusted within 7 days of the G61.8 registered date. These immunousuppressive agents including azathioprine, mycophenolate mofetil, methotrexate, tacrolimus, cyclosporine, cyclophosphamide, or rituximab. Third, all patients who had previously been labelled or had their codes changed to G60 (hereditary and idiopathic neuropathy), G62 (other polyneuropathies), G63 (polyneuropathy in diseases classified elsewhere), or G64 (other disorders of the peripheral nervous system) between 1st January, 2016 to 30th June, 2021 were excluded from the study population. Fourth, individuals who had been diagnosed with cancer up to 5 years prior to their CIDP diagnosis were excluded from the study. Finally, patients labeled as C88 (malignant immunoproliferative disease including Waldenstrom macroglobulinaemia and other diseases) and C90 (multiple myeloma and malignant plasma cell neoplasm) during above study period were excluded to eliminate patients with paraproteinemic neuropathy (13).

In reporting this study the authors followed the Strengthening the Reporting of Observational studies in Epidemiology (STROBE) guidelines.

### 2.3 Clinical characteristics

Patients with CIDP were surveyed to obtain information regarding their treatment history and comorbidities. The Elixhauser Comorbidity Index (ECI) score was used to measure the burden of comorbid diseases (14), but certain conditions were excluded from the analysis. Specifically, acquired immunodeficiency syndrome, human immunodeficiency virus infection, drug abuse, psychosis, and depression were excluded because of legal access restrictions for human rights. Additionally, codes related to solid tumors without metastasis, metastatic cancer, and lymphoma were excluded, as they were outcomes of the study. A comprehensive list of KCD-7,8 diagnosis codes used to calculate ECI scores is presented in Supplementary Table S1. The ECI score was then divided into three categories based on its distribution in the study cohort: “score = 0,” “score = 1,” and “score = ≥2.” These categories were used for descriptive and multivariable analyses.

### 2.4 Cancer incidence

To identify incident cancer cases, we searched for KCD-7,8 codes recorded as the principal diagnosis. Supplementary Table S2 presents the diagnostic codes used to identify cancer patients; secondary and benign tumors were excluded from the analysis. We included only the first cancer diagnosis after the date of CIDP diagnosis as an event. As mentioned previously, patients with C88 or C90 were not originally included in the study population and were excluded from the analysis of cancer incidence. All patients with CIDP were followed up from the date of diagnosis until cancer diagnosis, emigration, death, or the end of the observation period (July 30, 2021), whichever occurred first. We calculated standardized incidence ratios (SIRs) as the ratio of the observed to the expected number of cancer events in the general population. The number of expected cancer events was obtained by multiplying the age- and sex-specific incidence rates in the general Korean population with the number of person-years at risk for each age and sex stratum in the CIDP population (15). As the age-, sex-, and cancer-specific incidence rates for 2020 and 2021 in the general Korean population were not available, we used the average incidence rates for 2016–2019.

### 2.5 Statistical analysis

The 95% confidence intervals (CIs) of the SIRs were estimated assuming that the number of observed cancer events followed a Poisson distribution. If the observed number of events was less than five, the 95% CI of the SIRs was estimated using the exact method. A Cox proportional hazards model was used to investigate the factors (age, sex, treatment, or comorbities) associated with cancer incidence in patients with CIDP. The above proportional hazards assumption for the Cox model was verified using the Schoenfeld residual test. All results are presented as 95% CIs. Analyses were conducted using R for Windows, version 4.2.0.

## 3 Results

### 3.1 Demographics and clinical characteristics

**Figure 1:**
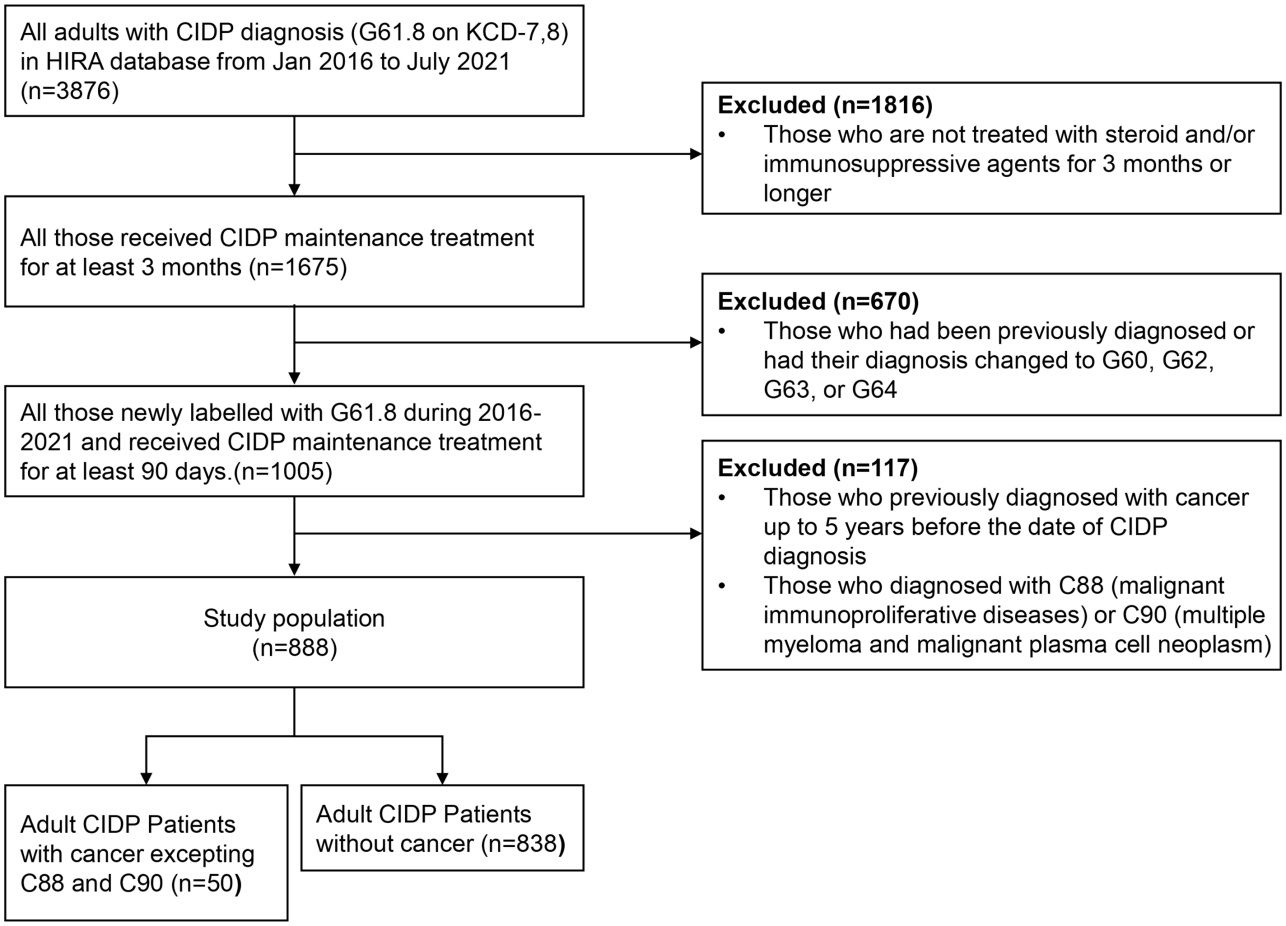
Flowchart of the study population selection. CIDP, chronic inflammatory demyelinating polyneuropathy; KCD-7,8, seventh and eighth revision of Korean Standard Classification of Disease; HIRA, Korean Health Insurance Review and Assessment Service.

Between January 1, 2016 and July 30, 2021, 3,876 patients with CIDP were assigned with the diagnostic code G61.8 (Figure 1). Among these patients, 1,675 were evaluated after adjusting for long-term immunosuppressive treatment for ≥3 months. During the study period, 1,005 patients with CIDP were initially assigned and maintained as having a diagnostic code of G61.8, whereas the remaining 670 patients had been previously classified with other diagnoses or had their diagnosis changed to G60, G62, G63, or G4 codes. Among the 1,005 patients with CIDP, 99 were excluded from the study because of a previous diagnosis of cancer, and 18 were subsequently diagnosed with C90 (multiple myeloma and malignant plasma cell neoplasms) or C88 (malignant immunoproliferative diseases). Of the remaining 888 patients, 50 (5.63% of enrolled CIDP patients) were newly diagnosed with cancer during the study period. Among them, 32 (64%) cancer patients with CIDP were ≥ 60 years old (Table 1). Further, 36 (72%) patients were male, and 44 (88.00%) were initially administered prednisolone as maintenance therapy for CIDP. Additionally, 13 (26%) patients were treated with other immunosuppressive agents, including azathioprine, mycophenolate mofetil, methotrexate, tacrolimus, cyclosporine, cyclophosphamide, or rituximab. Azathioprine was the most frequently administered drug (11 patients). Furthermore, 37 (74%) patients with cancer were classified into “≥2” group based on ECI score. The proportion of patients in the older age group was higher in the cancer group (64% of 50 patients) than in the non-cancer (47.4% of 838 patients) group among patients with CIDP (Table 1, *p* < 0.010). Other factors, including sex, medication history, and ECI score, were not related to cancer occurrence in the two groups. Older age was the only factor associated with an increase in cancer occurrence among all patients with CIDP (Table 2).

**Table 1:** Demographic and clinical characteristics of adult CIDP patients in South Korea according to HIRA database between 2016–2021.

Characteristic	All patients with CIDP (*n* = 888)	Without cancer (*n* = 838)	With cancer^*^ (*n* = 50)	*p*-value
Age at diagnosis, *n* (%)				0.010
less than 40	112 (12.61)	111 (13.20)	1 (2.00)	
40–59	347 (39.08)	330 (39.40)	17 (34.00)	
≥60	429 (48.31)	397 (47.40)	32 (64.00)	
Male sex, *n* (%)	578 (65.09)	542 (64.70)	36 (72.00)	0.066
Steroid as maintenance therapy, *n* (%)	698 (78.60)	654 (78.00)	44 (88.00)	0.125
Oral immunosuppressive agents as maintenance therapy, *n* (%)^†^	222 (25.00)	209(24.90)	13 (26.00)	0.354
Azathioprine	167	156	11	
Mycophenolate mofetil	47	46	1	
Methotrexate	10	9	1	
Tacrolimus	16	16	0	
Cyclosporine	9	8	1	
Cyclophosphamide	1	1	0	
Rituximab	3	1	2	
Treatment with IVIG, *n* (%)	290 (32.66)	271 (32.30)	19 (36.00)	0.540
Plasma exchange, *n* (%)	23 (2.59)	21 (2.50)	2 (4.00)	0.894
Elixhauser Comorbidity Index Score^‡^, *n* (%)				0.510
0	84 (9.50)	78 (9.30)	6 (12.00)	
1	164 (18.47)	157 (18.70)	7 (14.00)	
≥2	640 (72.07)	603 (72.00)	37 (74.00)	

^*^Malignant immunoproliferative diseases (C88) and multiple myeloma and malignant plasma cell neoplasm (C90) was not included. ^†^Treatment by multiple drugs was reflected. ^‡^Modified to exclude cancer-solid tumor without metastasis, metastatic cancer, lymphoma. CIDP, Chronic inflammatory demyelinating polyneuropathy; IVIG, Intravenous immunoglobulin.

**Table 2:** Hazard ratios for cancer incidence among adult CIDP patients according to HIRA database between 2016–2021.

Variable	Univariate analysis			Multivariate analysis		
	HR	(95% CI)	*p*-value	HR	(95% CI)	*p*-value
**Age at diagnosis (years)**						
Less than 40	1(reference)			1(reference)		
40–59	7.93	(1.08–58.44)	0.04^*^	8.31	(1.12–61.58)	0.04^*^
≥60	10.90	(1.50–79.24)	0.01^*^	11.83	(1.61–86.98)	0.02^*^
**Sex (male/female)**	1.45	(0.78–2.81)	0.06	1.47	(0.76–2.85)	0.07
**Treatment with IVIG (yes/no)**	0.82	(0.48–1.40)	0.47	0.80	(0.46–1.37)	0.41
**Plasma exchange (yes/no)**	1.11	(0.27–4.54)	0.88	1.30	(0.31–5.55)	0.72
**Elixhauser comorbidity index score**^†^						
0	1(reference)			1(reference)		
1	0.65	(0.26–1.64)	0.36	0.56	(0.22–1.43)	0.23
≥2	0.77	(0.36–1.62)	0.49	0.56	(0.26–1.20)	0.14

CIDP, Chronic inflammatory demyelinating polyneuropathy; HR, Hazard ratio; CI, Confidence interval; IVIG, Intravenous immunoglobulin. ^*^Statistically significant, *p* < 0.05. ^†^Modified to exclude cancer-solid tumor without metastasis, metastatic cancer, lymphoma.

### 3.2 Cancer incidence in patients with CIDP

The number of expected cancer events obtained by multiplying the age- and sex-specific incidence rates in the CIDP population during study period was 17.78 (2% of incidence rate), but the actual observed events number was 50 (5.63% of incidence rate) with an SIR of 2.83 (CI: 1.89–4.39) (Table 3). The most prevalent age group among CIDP patients with cancer was in their 70s, with a total of 16 individuals. Groups of four decades between 40 and 79 years of age had higher than one on the minimum value of SIRs in cancer incidence. Malignancy of digestive organs (C15-25) was most common in patients with CIDP (Table 4, 13 patients, 1.46% of 888 CIDP patients). Malignancy of lymphoid, hematopoietic and related tissues, except malignant immunoproliferative diseases, multiple myeloma, and plasma cell neoplasms (C81-96 excepting C88 and C90, six patients, 0.68%) was noted in six patients with the highest SIR (8.51, CI: 4.18–19.83). Their SIRs compared to expected numbers was highest in both male (7.56, CI: 3.90–20.97) and female (10.50, CI: 2.09–33.65) groups. All six patients were diagnosed with non-Hodgkin lymphoma: three males and one female with non-follicular lymphoma (C83), one female with mature T/NK-cell lymphoma (C84), and one male with unspecified types of non-Hodgkin lymphoma (C85). Melanoma and other skin (C43-44) neoplasm was also commonly observed (two patients, 0.25%) than expected number; however, these two patients are female (9.92, CI: 1.20–35.85). Meanwhile, lung cancer (C33-34) was observed in eight patients (0.88%) and the minimum SIR was higher than one in male group (2.51, CI: 1.05–5.18); not in the female group (4.30, CI: 0.52–15.52). Malignancy of male genital organs (C60-63) also had higher than one of the minimum SIR (2.65, CI: 1.01–5.81), whereas malignancy of female genital organs wasn’t (5.03, CI: 0.61–18.15).

**Table 3:** Standardized incidence ratios of any cancer stratified by age in adult CIDP patients in South Korea according to HIRA database between 2016–2021.

Age at CIDP diagnosis	No. of patients with CIDP	Cancers		SIR (95% CI)
		No. observed	No. expected	
All age	888	50 (5.63%)	17.78 (2.00%)	2.83 (1.89–4.39)^*^
20–29 years	39	0	0.04	0 (0.00–82.03)^†^
30–39 years	73	1	0.32	3.14 (0.08–17.49)
40–49 years	146	8	1.26	6.35 (2.43–11.48)^*^
50–59 years	202	10	3.06	3.30 (1.56–6.57) ^*^
60–69 years	211	12	4.79	2.56 (1.31–5.17)^*^
70–79 years	167	16	6.57	2.48 (1.42–4.31)^*^
>80 years	50	3	1.73	1.73 (0.36–5.06)

^*^Indicates the minimum value of SIR > 1. ^†^No case observed. CIDP, chronic inflammatory demyelinating polyneuropathy; CI, confidence interval; SIR, Standardized incidence ratio.

**Table 4:** Standardized incidence ratios of cancer types stratified by sex in adult CIDP patients in South Korea according to HIRA database between 2016–2021.

Cancer site (KCD-7 codes)	Male (*n* = 578)			Female (*n* = 310)			Total (*n* = 888)		
	Cancers		SIR (95% CI)	Cancers		SIR (95% CI)	Cancers		SIR (95% CI)
	No. observed	No. expected		No. observed	No. expected		No. observed	No. expected	
All cancer	36	14.37	2.52(1.53–4.11)^*^	14	5.04	2.78(1.39–4.73)^*^	50	17.78	2.83(1.89–4.39)^*^
Digestive organs (C15-25)	12	6.79	1.77(0.96–2.99)	1	1.72	0.58(0.01–3.25)	13	8.51	1.53(0.86–2.54)
Lung (C33-34)	6	2.39	2.51(1.05–5.18)^*^	2	0.47	4.30(0.52–15.52)	8	2.85	2.81(1.32–5.29)^*^
Melanoma and other skin (C43-44)	0	0.33	0^†^	2	0.20	9.92(1.20–35.85)^*^	2	0.53	3.79(0.46–13.68)
Breast (C50)	0	0.01	0^†^	3	0.90	3.33(0.69–9.75)	3	0.91	3.29(0.68–9.62)
Female genital organs (C51-58)				2	0.40	5.03(0.61–18.15)			
Male genital organs (C60-63)	5	1.89	2.65(1.01–5.81)^*^						
Urinary tract (C64-68)	2	0.94	2.13(0.26–7.71)	0	0.16	0^†^	2	1.1	1.82(0.22–6.59)
Thyroid and other endocrine glands (C73-75)	1	0.44	2.27(0.06–12.66)	2	0.72	2.77(0.34–10.00)	3	1.16	2.58(0.53–7.54)
Lymphoid, haematopoietic and related tissue (C81-96) excepting malignant immunoproliferative diseases (C88) and multiple myeloma and malignant plasma cell disorders (C90, *n* = 15)	4	0.53	7.56(3.90–20.97)^*^	2	0.19	10.50(2.09–33.65)^*^	6	0.72	8.51(4.18–19.83)^*^

^*^Indicates the minimum value of SIR > 1. ^†^No case was observed. CIDP, chronic inflammatory demyelinating polyneuropathy; KCD-7, 7th revision, Korean version of the International Classification of Diseases; CI, confidence interval; SIR, standardized incidence ratio.

## 4 Discussion

In this nationwide cohort study, we compared the incidence of cancer in patients with CIDP with that of expected number calculated for the general Korean population. The study outcome indicated 2.83-fold higher incidence of cancer in patients with CIDP than the expected cancer incidence rates. The pathogeneses of CIDP and cancer are largely unknown; however, they are believed to be multifactorial diseases related to genetic, environmental, and endogenous factors. Age is recognized as a risk factor for both cancer and CIDP (16, 17). This study demonstrates a higher incidence of cancer in older CIDP patients, along with elevated SIRs for any cancer compared to same age group without CIDP. There seems to be a sex difference in the association between CIDP and the risk of certain types of cancers, although the number of incident events was small.

The most concerning issue in the context of prolonged administration of immunosuppressants is their potential impact on the development of cancer. Previous studies have showed controversial results regarding the association between azathioprine and the occurrence of cancer among its recipients (18–21). The aforementioned studies have indicated that lymphoid tissue or skin cancer are most strongly associated with azathioprine (19–21). These malignancies also exhibited higher incidence rate among the CIDP patients in this study. However, due to insufficient patient numbers in this study, a comprehensive analysis of the risks associated with each immunosuppressant could not be conducted. Therefore, it is not possible to conclude that any specific medication acts as a risk factor for cancer in CIDP. It is deemed necessary that subsequent studies with a larger cohort of patients include analyses related to this matter. In particular, tacrolimus is more commonly used in immune related neurologic disorders in East Asia, and there have been reports suggesting a greater association of this medication with cancer compared to other drugs (18, 22–24). Subsequent studies should consider these factors.

In this population-based study, a discernible increase in the incidence of hematological malignancies was observed among patients with CIDP. Specifically, non-Hodgkin lymphoma emerged as the predominant subtype, which is consistent with the findings of previous studies (6, 25). Although the precise mechanisms and causative factors have not been definitively established, a correlation between lymphomas and peripheral nervous system abnormalities has been consistently reported. Specifically, rare conditions, such as paraneoplastic neuropathies or neurolymphomatosis, affecting the peripheral and/or cranial nerves are known (26, 27). Additionally, there has been a focus on chemotherapy-induced peripheral neuropathy as a complication of lymphoma treatment (28). Autoantibody-mediated polyneuropathies or acquired inflammatory polyneuropathies in patients with lymphoma are considered evidence of a complex interrelationship between autoimmunity and lymphoma (25).

In this study, the incidence of skin cancer was higher in female patients with CIDP. Among CIDP patients with melanoma reported in a previous systematic review (6), the number of male patients was higher, when only cases where sex could be confirmed were considered (nine males, four females, and one unknown). This may be owning to a number of CIDP events associated with melanoma patients caused by melanoma treatment (6, 29, 30). In addition, it is important to consider that the incidence of skin cancer is lower in East Asian populations, including South Korea (31).

Previous case studies have reported patients with CIDP who were diagnosed with small cell lung carcinoma and exhibited anti-Hu and/or anti-CRMP paraneoplastic autoantibodies (32, 33). While our study also found a higher risk of lung cancer in patients with CIDP, this study alone is insufficient to establish a direct link between paraneoplastic syndrome and increased cancer incidence rates in patients with CIDP. Specifically, a significant increase in the incidence of lung cancer was observed only in male patients, and the specific subtype of small cell lung cancer could not be confirmed using the HIRA database. Furthermore, the smoking history of the patients was not recorded in this database. Additionally, this study identified a higher incidence of genital cancer among male patients with CIDP. The outcomes of male genital cancers mentioned above might be related to smoking history, and further detailed investigation is needed to demonstrate the relationship between these cancers and CIDP.

This study had several limitations. First, the higher cancer incidence observed among patients with CIDP in this study might biased in several perspectives. This study, conducted utilizing big data, employs operational definitions due to the imperfectly curated nature of the information sources regarding the target disease group. Therefore, it is essential to consider the possibility of selection and information bias that may arise while using operational definitions. To be more specific, while establishing our research cohort, we extensively utilized the C codes to exclude paraproteinemic neuropathy, however, there remains a possibility that monoclonal gammopathy of undetermined significance (MGUS) was not effectively filtered out. Even though cases where MGUS progressed to a malignancy falling under the C codes would have been excluded, the potential occurrence of neuropathy due to MGUS must be considered as a potential source of bias. Expanding on these perspectives, it’s possible that this operationally defined CIDP cohort is just excluded individuals receiving immunoglobulin as initial treatment in CIDP, thus might not adequately represent the CIDP population. Especially considering the reality that a significant number of diagnoses of CIDP change after the initial diagnosis (34), caution is warranted in interpreting this study. Furthermore, although patients were analyzed by categorizing them into ECI items, it’s important to acknowledge that individuals may simultaneously have multiple health issues, thus confounding bias cannot be entirely excluded. Second, the number of patients with cancer and CIDP enrolled in this study was too small to be generalized to the clinical setting. This may be owning to the strict application of operational definitions. In particular, the fact that comorbidities measured by the ECI score are not risk factors for overall cancer incidence may be due to the small number of enrolled patients. We primarily described cancer incidences for which the minimum value of SIR was greater than one in this study. However, due to the very small number of events, it does not necessarily mean that the high SIR values of those cancer types with minimum values lower than one are meaningless. Third, the study period might have temporal ambiguity. In this study, all cancer-diagnosed patients were previously treated for CIDP, but the data covers only five years. For this reason, the influence of CIDP on cancer occurrence might not be directly related to CIDP itself or its treatment as initially expected. Additionally, data from 2020 to 2021 were not included to measure the expected number of cancer events in the general Korean population. This omission might have made the risk evaluation less accurate, and this ambiguity should be considered when interpreting the results of this study. Nevertheless, considering that the annual cancer incidence rate in South Korea does not change significantly, this may not result in a significant error compared to what was initially presumed.

## 5 Conclusion

To the best of our knowledge, this is the first population-based cohort study to examine the cancer risk in patients with CIDP. We found that the actual cancer incidence was 2.83-fold higher in patients with CIDP than the expected cancer incidence rate and that some cancers had different risks according to sex. These results highlight the importance of monitoring the occurrence of cancer in the management of patients with CIDP. Further epidemiological and clinical investigations including longer study period with prospective design of CIDP are required to replicate this study.

## Data Availability

The original contributions presented in the study are included in the article/Supplementary material, further inquiries can be directed to the corresponding author.
