# Supplementary material for: Incidence of cancer in chronic inflammatory demyelinating polyneuropathy: a nationwide cohort study in South Korea
Source: Front Neurol. 2024 Aug 29;15:1456835. doi: 10.3389/fneur.2024.1456835 (PMC11390450; doi:10.3389/fneur.2024.1456835)
Supplement: Supplementary file 1 [file Table_1.DOCX]

Supplementary Material

# Supplementary Tables

**Supplementary Table 1.** Calculation of the Elixhauser Comorbidity Index Score using the Korean Standard Classification of Disease code, 7^th^ and 8^th^ revision (KCD-7,8; Korean version of the International Classification of Diseases, 10^th^ and 11^th^ revision).

| **Comorbidity** | **KCD-7,8 (ICD-10,11)** |
| --- | --- |
| Congestive heart failure | I09.9, I11.0, I13.0, I13.2, I25.5, I42.0, I42.5-I42.9, I43.x, I50.x, P29.0 |
| Cardiac arrhythmias | I44.1-I44.3, I45.6, I45.9, I47.x-I49.x, R00.0, R00.1, R00.8, T82.1, Z45.0, Z95.0 |
| Valvular disease | A52.0, I05.x-I08.x, I09.1, I09.8, I34.x-I39.x, Q23.0-Q23.3, Z95.2-Z95.4 |
| Pulmonary circulation disorders | I26.x, I27x, I28.0, I28.8, I28.9 |
| Peripheral vascular disorders | I70.x, I71.x, I73.1, I73.8, I73.9, I77.1, I79.0, I792, K55.1, K55.8, K55.9, Z95.8, Z95.9 |
| Hypertension | I10.x, I11.x-I13.x, I15.x |
| Paralysis | G04.1, G11.4, G80.1, G80.2, G81.x, G82.x, G83.0-G83.4, G83.9 |
| neurodegenerative disorders | G10X-G13.x, G20.x-G22.x, G25.4, G25.5, G31.2, G31.8, G31.9, G32.x, G35.x-G37.x, G40x, G41x, G93.1, G93.4, R47.0, R56.x |
| Chronic pulmonary disease | I27.8, I27.9, J40.x-J47.x, J60.x-J67.x, J68.4, J70.1, J70.3 E10.0, E10.1, E10.9, E11.0, E11.1, E11.9, E120, E12.1, E129, E13.0, E13.1, E13.9, E14.0, E14.1, E14.9 |
| Diabetes | E10.2-E10.8, E11.2-E11.8, E12.2-E12.8, E13.2-E13.8, E14.2-E14.8 |
| Hypothyroidism | E00.x-E03.x, E89.0 |
| Renal failure | I12.0, I13.1, N18.x, N19.x, N25.0, Z49.0-Z492, Z94.0,Z99.2 |
| Liver disease | B18.x, I85.x, I86.4, I98.2, K70.x, K71.1, K71.3-K71.5, K71.7, K72.x-K74.x, K76.0, K76.2-K76.9, Z94.4 |
| Peptic ulcer disease, excluding bleeding | K25.7, K25.9, K26.7, K26.9, K27.7, K27.9, K28.7, K28.9 |
| Rheumatoid arthritis/collagen vascular diseases | L94.0, L94.1, L94.3, M05.x, M06x, M08.x, M12.0, M12.3, M30.x, M31.0-M31.3, M32.x-M35.x, M45.x, M46.1, M46.8, M46.9 |
| Coagulopathy | D65-D68.x, D69.1, D69.3-069.6 |
| Obesity | E66.x |
| Weight loss | E40.x-E46.x, R63.4, R64 |
| Deficiency anemia | D50.8, D50.9, D51.x-D53.x |
| Fluid and electrolyte disorders | E22.2, E86.x, E87.x |
| Blood loss anemia | D50.0 |
| Alcohol abuse | E52, G62.1, I42.6, K29.2, K70.0, K70.3, K70.9, I51.x, Z50.2, Z71.4, Z72.1 |

KCD, Korean version of the International Classification of Diseases; ICD, International Classification of Diseases.

**Supplementary Table 2.** Cancer sites and used the Korean Standard Classification of Disease code, 7^th^ and 8^th^ revision for identifying the incidence of cancer. (KCD-7,8; Korean version of the International Classification of Diseases, 10^th^ and 11^th^ revision).

| **Cancer site** | **KCD-7,8 (ICD-10, 11)** |
| --- | --- |
| digestive organs | C15-25 |
| lung | C33-34 |
| Melanoma and other skin | C43-44 |
| breast | C50 |
| female genital organs | C51-58 |
| male genital organs | C60-63 |
| urinary tract | C64-68 |
| thyroid and other endocrine glands | C73-75 |
| lymphoid, haematopoietic and related tissue | C81-96 |
| *Malignant immunoproliferative diseases^*^* | *C88* |
| *Multiple myeloma and malignant plasma cell neoplasms^*^* | *C90^*^* |

KCD, Korean version of the International Classification of Diseases; ICD, International Classification of Diseases.

^*^Those who were labelled with C88 or C90 were excluded from the study population.
